# P-2359. CLEAR Hepatitis C – Development and impact of a regional collaborative to facilitate identification and treatment of patients with Hepatitis C

**DOI:** 10.1093/ofid/ofae631.2510

**Published:** 2025-01-29

**Authors:** Andrew Kessell, Madonna A Biritwum

**Affiliations:** FirstHealth Moore Regional Hospital, Pinehurst, North Carolina; FirstHealth, Southern Pines, North Carolina

## Abstract

**Background:**

Despite advancements in therapy, hepatitis C continues to significantly impact public health and individual patients. Untreated hepatitis C can lead to liver failure and death. The introduction of oral therapies provides an opportunity to cure patients’ hepatitis C and limit the spread. However, challenges persist in the identification of patients, access to specialists trained in the treatment of hepatitis C and the medication therapy costs. FirstHealth of the Carolinas (FHC) developed partnerships and care pathways within our regional medication community including employed and non-employed physician practices. The principles of the care pathway were, CLEAR Hepatitis C (Figure 1). The purpose of our study was to evaluate the outcomes associated with a comprehensive regional approach to identification and treatment of patients with hepatitis C.

CLEAR Hepatitis C acronym

**Figure:**
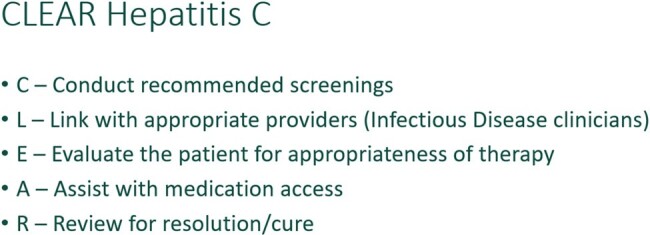

Outlines care pathway developed for the treatment of hepatitis C

**Methods:**

A retrospective study was conducted at FHC reviewing outcomes associated with the CLEAR Hepatitis C initiative undertaken from September 2023 through April 2024. Data evaluated included: hepatitis C antibody screens conducted by primary care providers, Infectious Disease clinic referrals for hepatitis C treatment, oral therapies prescribed by Infectious Disease providers, patients assisted with medication therapy by specialty pharmacists and pharmacy technicians and patients receiving a curative diagnosis.

CLEAR Hepatitis Results

**Figure:**
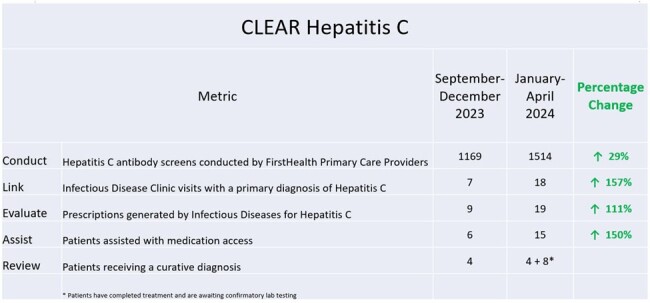

Results associated with each step of the care pathway.

**Results:**

Primary care provider education and promotion of CLEAR Hepatitis C initiative commenced in November 2023. Comparing the fourth month periods of September-December of 2023 to January-April 2024: the total hepatitis C antibody screens conducted increased from 1169 to 1514 (p < 0.05); Infectious Disease clinic visits with a primary diagnosis of hepatitis C increased from 7 to 18; Prescriptions from Infectious Disease providers increased from 9 to 19; Patients assisted with medication access increased from 6 to 15. During the study period, 4 patients received a curative diagnosis. An additional 8 patients have completed treatment and are awaiting confirmatory lab testing (Figure 2).

**Conclusion:**

Development of a collaborative, integrated hepatitis C care pathway within our regional medical community successfully increased the number of patients identified and treated for hepatitis C in our region.

**Disclosures:**

All Authors: No reported disclosures

